# Impact of cancer outcome data source on the diagnostic accuracy of ovarian cancer prediction models: a primary care cohort study

**DOI:** 10.1136/bmjph-2025-004229

**Published:** 2026-03-25

**Authors:** Yi Ting Yu, Fiona M Walter, Kirsten D Arendse, Garth Funston

**Affiliations:** 1 Centre for Cancer Screening, Prevention and Early Diagnosis, Wolfson Institute of Population Health, Queen Mary University of London, London, UK; 2 Department of Public Health and Primary Care, University of Cambridge, Cambridge, UK

**Keywords:** Public Health, Risk Assessment, Preventive Medicine

## Abstract

**Objectives:**

Electronic health records are widely used to develop diagnostic prediction models for cancer. Some studies use cancer registry (CR) data, the gold standard for cancer case recordings, whereas others rely on data from alternative healthcare sources. We aimed to evaluate the impact of using CR and non-CR data sources on the diagnostic accuracy of the Ovatools ovarian cancer (OC) risk prediction model.

**Methods:**

Retrospective cohort study using linked Clinical Practice Research Datalink (CPRD), hospital episodic statistics (HES) and CR data from women tested for cancer antigen 125 (CA125) in England (1 May 2011–31 December 2017). Ovatools model performance and diagnostic accuracy were compared when different data sources were used, alone and in combination, to identify the outcome, OC diagnosis in the year after CA125 testing. Threshold accuracy was measured at the National Institute for Health and Care Excellence ≥3% risk threshold.

**Results:**

Among 340 769 CA125-tested women, OC incidence within 12 months was highest when using HES data (0.84%), compared with CR (0.75%) and CPRD (0.65%). Area under the curve was highest when using CR alone (0.924) and lower using CPRD (0.903) or CR+CPRD+HES (0.892). At a ≥3% risk threshold, sensitivity was highest when using CR data (73.2%) and lower using CPRD (68.8%). The positive predictive value was lowest using CPRD (13.8%) and highest using CPRD+CR+HES (19.4%).

**Conclusion:**

Using an OC exemplar, we found moderate variation in model performance and threshold accuracy when different data sources were used to define cancer. To ensure cancer prediction models perform as expected in real world clinical practice, gold standard data sources, such as CR data, should be used for model development and validation.

**WHAT IS ALREADY KNOWN ON THIS TOPIC**

- Cancer prediction models in the UK are often developed and evaluated using large routinely collected healthcare data sources.
- Accurate identification of cancer cases is essential for robust model development and evaluation.
- Prediction modelling studies have used a range of data sources to identify cancer data and the impact of this is unclear.

**WHAT THIS STUDY ADDS**

- This is the first study investigating the impact of different cancer data sources in model performance and diagnostic accuracy of a cancer prediction model.
- Our findings illustrate the source of cancer data results in variable estimates of model performance and diagnostic accuracy.

**HOW THIS STUDY MIGHT AFFECT RESEARCH, PRACTICE OR POLICY**

- Our study suggests that routine data sources such as General Practice records may overestimate or underestimate cancer incidence or experience a delay in capturing diagnosis dates, leading to an invalid assessment of model performance and threshold accuracy.
- Wherever possible, studies should use gold standard sources for cancer outcomes, such as cancer registry data, to minimise the risk of misclassification bias.

## Introduction

 Cancer is the leading cause of death globally, responsible for an estimated 10 million deaths in 2020.^1^ In the UK, cancer accounts for one in four deaths,^2^ with survival rates lower than other European countries.^3^ Within the National Health Service (NHS), people with cancer typically first present to their general practitioner (GP) with non-specific symptoms.^3^ As cancer is relatively rare in the UK primary care setting, this diagnostic uncertainty poses a considerable challenge for GPs. Screening supports early detection for certain cancers, but it is limited to a few types. For rarer cancers, screening is difficult to justify due to lack of cost-effectiveness and potential harms. Early identification of symptomatic cancer is essential to improve outcomes.

Predictive models offer a promising approach to early detection. Clinical decision support tools integrating risk factors, symptoms and test results can estimate cancer risk and guide further investigations or referral. The UK’s electronic health record (EHR) systems, including Clinical Practice Research Datalink (CPRD)^4^ and QResearch,^5^ are valuable resources of data for developing and validating predictive models for rare cancers, where prospective studies are costly and time-consuming.

Examples of UK-developed tools are QCancer^6^ and Risk Assessment Tools,^7 8^ which have been integrated into NHS primary care systems or made available online to support cancer risk assessment. Recently, complex artificial intelligence models using EHR have been developed for multiple cancer types, illustrating potential to support early cancer diagnosis.^9^ However, EHRs have limitations, particularly regarding the accuracy of clinical event incidence dates such as cancer diagnosis.^10^ Misclassifications can lead to overestimations or underestimations in risk prediction models; thus, models could potentially fail to perform similarly in real-world practice.^11^ Selecting an appropriate data source is therefore critical, especially to define rare cancers. Cancer registries (CRs), such as National Cancer Registration and Analysis Service (NCRAS) in England, are considered the gold standard for cancer case ascertainment as they consolidate multiple data sources to identify and verify cancer cases and provide detailed morphological and staging data.^12^,^15^ However, many previous cancer prediction modelling studies have used only non-CR data sources,^16 17^ while some have used a combination of CR and non-CR data sources to define outcomes.^18 19^

Researchers have suggested the utilisation of non-CR data may impact model performance,^11 14^ but evidence on how data sources impact predictive model diagnostic accuracy and performance remains limited. This study examined how using different data sources to define ovarian cancer (OC) diagnosis affects estimates of the performance of an existing OC prediction model.^18^

## Methods

### Design and data source

This study built on the design used in an external validation study of the Ovatools model, a prediction model incorporating cancer antigen 125 (CA125) level and age to predict OC risk.^20^ The UK’s CR database (National Cancer Registration and Analysis Service, NCRAS) was used to determine cancer outcomes in Ovatools development and validation studies.^18 20^

This retrospective cohort study used data from CPRD Aurum data linked with Hospital Episode Statistics Admitted Patient Care (HES APC), and NCRAS.^4 12 21^ CPRD is a primary care EHR of anonymised patient information collected from GPs in the UK using Egton Medical Information Systems software and is broadly representative of the UK population.^4^ It includes demographic data, medical history, medications, laboratory investigations and specialist referrals for over 60 million patients.^4^ HES APC provides hospital-level electronic records, including clinical, demographic and administrative data for NHS hospital admissions.^21^ NCRAS collects longitudinal data on all people with cancer in England, including data on tumour morphology, histology, incidence date and stage, and is considered the gold standard for cancer case identification in epidemiological studies.^12 20^

### Population

The baseline cohort was drawn from that used in a previous study.^20^ We included women with a valid CA125 measurement recorded in CPRD between 1 May 2011 and 31 December 2017. CA125 tests were considered valid if they were not missing and above 0 U/mL. The first CA125 test during the study period was the index test. We excluded women aged <18 years at the time of the index test, those with previous CA125 tests in the year before their index test and those with a history of OC or borderline ovarian tumours in any data source.

### Outcome

The primary outcome was OC, as defined by the International Classification of Disease (ICD) 10th edition derived by the WHO and Federation of International Gynaecological and Obstetrics. This included invasive OC (ICD-10 codes: C56, C57.0, C48.1, C48.2) and borderline ovarian tumours (ICD-10 code: D39.1).^22^ Borderline tumours were included as they could not always be distinguished from invasive cancers in some data sources (CPRD, HES APC).

Three English EHR databases were used to identify codes for OC incidence, namely: (1) CPRD, (2) HES APC and (3) NCRAS. NCRAS used ICD-02/03 codes, with specification of tumour morphology published previously.^20^ ICD-10 codes were used to identify OC from HES APC. In CPRD, OC records were determined using a Systematised Nomenclature of Medicine Clinical Terms (SNOMED CT) code list defined by KDDA AND GF (online supplemental Table 1). OC records from HES APC were defined using ICD-10 codes as above.

Five primary outcomes were created for analysis to define OC incidence using different data sources: (1) CPRD, (2) HES APC, (3) NCRAS, (4) CPRD and NCRAS, and (5) CPRD, HES APC and NCRAS. Combinations focused on those including NCRAS as the intention was to evaluate how adding routine datasets altered the diagnostic accuracy relative to the gold standard for cancer records of NCRAS. In each data source, OC incidence was defined as a record within 12 months from the index CA125 test date. The proportion of OC in 12 months was summarised with 95% CIs calculated with exact binomial methods.

Sensitivity analyses were conducted using OC outcomes within 6 and 18 months after CA125. Outcomes based on a single data source were defined using that dataset alone, without incorporating information from other sources. Where multiple data sources were used to identify cancers, the earliest date of diagnosis across those datasets was used. For example, when using HES+CPRD+NCRAS to identify cancers, the earliest date of diagnosis across those three datasets was used.

### Analysis

The original Ovatools study used logistic regression models to predict OC risk using continuous age and continuous CA125 values, both modelled with restricted cubic splines due to non-linear associations. We applied the same prespecified risk prediction model parameters and knot placements for CA125 and age reported in the development study.^18^ The model estimated log odds risk of any OC, transformed into probability. This was repeated for each OC outcome.

Model performance was measured using discrimination and calibration. Discrimination was assessed using the area under curve (AUC) with 95% CIs. Calibration was reported using slope, intercept and calibration-in-the-large, with 95% CIs. Threshold accuracy was evaluated at ≥3% risk of OC, a National Institute for Health and Care Excellence (NICE) recommended threshold for urgent cancer referral.^23^ At this cut-off, sensitivity, specificity, positive predictive value (PPV) and negative predictive value were measured with 95% CI. We also evaluated the diagnostic accuracy of the CA125 threshold (≥35 U/mL) and a ≥1% risk threshold for OC at 6, 12 and 18 months across all OC outcome definitions.

As follow-up time can impact test accuracy and cancer recordings can vary between data sources,^24^ we conducted sensitivity analyses measuring OC outcomes within 6 and 18 months. As a secondary analysis, we examined code-level concordance between CPRD and HES APC diagnostic codes, using NCRAS as the reference standard to assess for coding practice discrepancies.

We used data from a previous model validation study, for which sample size calculations have been published.^20^ All data management was conducted in R V.4.4.2.^25^ Statistical analysis was conducted in Stata V.18.5.^26^

### Patient and public involvement

Patients and members of the public were not involved in the design, analysis or interpretation of this study. The study used anonymised EHR data from routinely collected sources.

## Results

### Descriptive analysis

**Figure 1:**
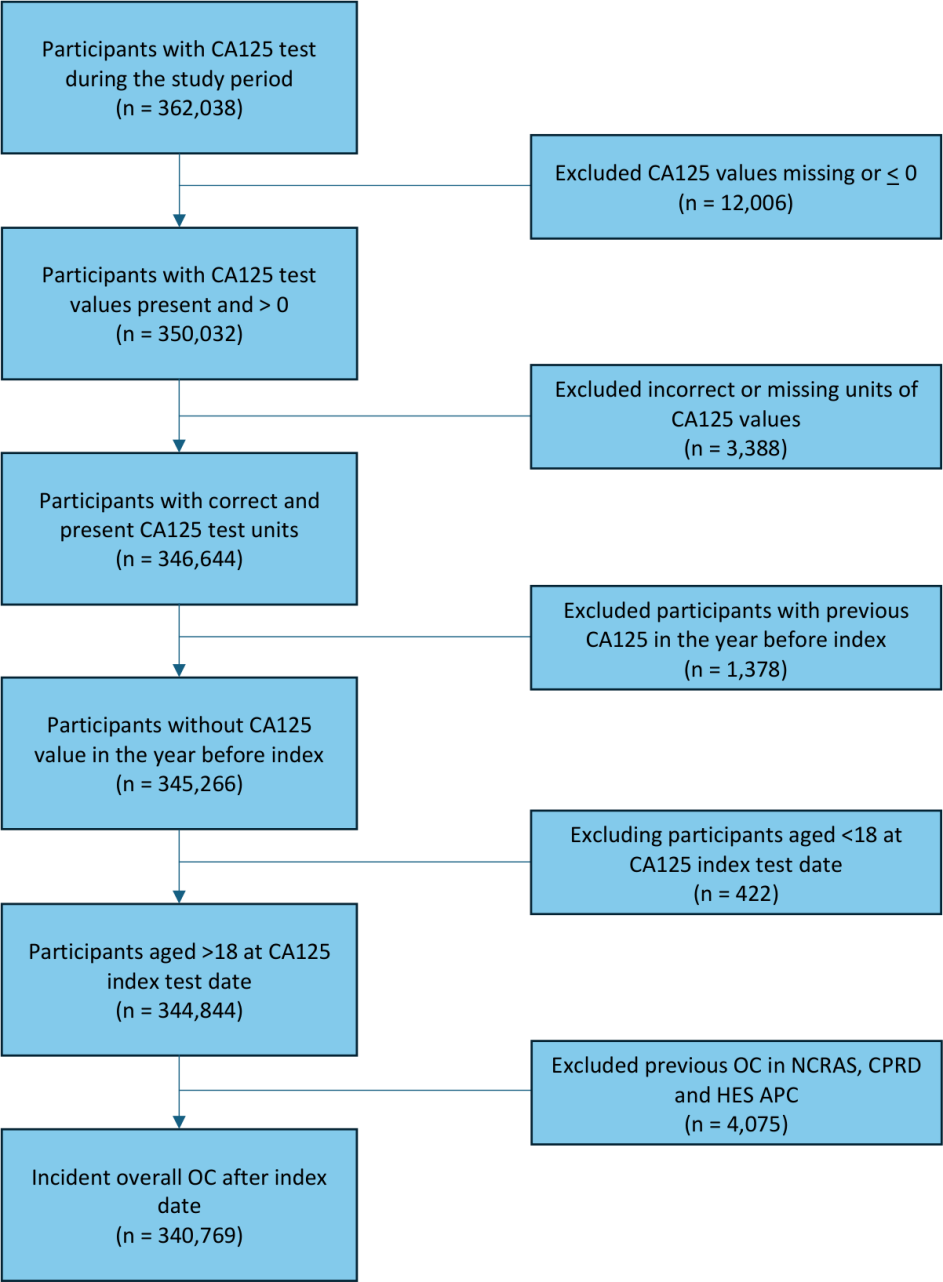
Flowchart for overall ovarian cancer patient cohort in NCRAS, CPRD and HES. CA125, cancer antigen 125; CPRD, Clinical Practice Research Datalink; HES APC, Hospital Episode Statistics Admitted Patient Care; NCRAS, National Cancer Registration and Analysis Service; OC, ovarian cancer;

During the study period, 346 644 participants had a valid CA125 test recorded in CPRD. Of these, 1378 were excluded due to a prior CA125 test within the preceding year, and 422 were excluded because they were aged <18 years at the index test date. 4075 were removed because of previous OC diagnosis before the index test. After applying these exclusion criteria, 340 769 participants remained in the final cohort (figure 1).

A total of 30 070 (8.82%) and 12 677 (3,72%) participants had an OC risk of ≥1% and ≥3%, respectively. The total number of participants with a CA125 ≥35 U/mL was 23 419 (6.87%) (table 1).

**Table 1:** Cohort characteristics, ovarian cancer incidence and CA125 and model risk distribution by data source

	Number (n)	Proportion (%), 95% CI
Total in cohort	340 759	NA
Age group (years)		
<30	18 275	5.36
30–39	39 529	11.60
40–49	85 125	24.98
50–59	77 429	22.72
60–69	56 899	16.70
≥70	63 502	18.64
Test distribution		
CA125 ≥35 U/mL	23 419	6.87
Ovatools risk ≥1%	30 070	8.82
Ovatools risk >3%	12 677	3.72
Ovarian cancer incidence within 12 months after index CA125 by data source		
CPRD	2535	0.74 (0.72 to 0.77)
NCRAS	2571	0.75 (0.73 to 0.78)
HES	2855	0.84 (0.81 to 0.87)
CPRD+NCRAS	3291	0.97 (0.93 to 1.00)
CPRD+NCRAS+HES	3761	1.10 (1.07 to 1.14)

CA125, cancer antigen; CPRD, Clinical Practice Research Datalink; HES, Hospital Episode Statistics; NA, not assessed; NCRAS, National Cancer Registration and Analysis Service.

Using individual sources of outcome data, the highest proportion of OC incidence within 12 months was recorded using HES APC (0.84%), followed by NCRAS (0.75%) and CPRD (0.74%) (table 1). Using combined data sources increased overall OC incidence: CPRD+NCRAS had an OC incidence of 0.97% and CPRD+NCRAS+HES APC had an incidence of 1.1% (table 1). Similar patterns were seen in sensitivity analyses using 6 and 18 months follow-up periods (online supplemental Table 2).

### Incidence of ovarian cancer by risk level

Among those with an OC risk of ≥3%, OC incidence within 12 months was lowest using CPRD (13.76%), compared with NCRAS (14.85%) and HES APC (16.42%) (table 2). This increased when using combined data sources: CPRD+NCRAS (17.6%) and CPRD+NCRAS+HES APC (19.4%) (table 2).

**Table 2:** Incidence of OC within 12 months after index CA125 by data source and risk level

Data source	Ovatools risk threshold	OC incidence, n (%)
CPRD	>3%	1743 (13.76)
	<3%	792 (0.24)
NCRAS	>3%	1881 (14.85)
	<3%	690 (0.21)
HES APC	>3%	2080 (16.42)
	<3%	775 (0.24)
CPRD+NCRAS	>3%	2230 (17.60)
	<3%	1061 (0.32)
CPRD+NCRAS+HES APC	>3%	2458 (19.40)
	<3%	1303 (0.40)

CA125, cancer antigen 125; CPRD, Clinical Practice Research Datalink; HES APC, Hospital Episode Statistics Admitted Patient Care; NCRAS, National Cancer Registration and Analysis Service; OC, ovarian cancer.

### Model performance

Good discrimination was observed when using all data sources. The AUC was highest with NCRAS (0.924, 95% CI 0.917 to 0.931), compared with CPRD alone (AUC 0.903, 95% CI 0.895 to 0.910) or in combined data source CPRD+NCRAS (AUC: 0.902; 95% CI 0.895 to 0.908) and CPRD+NCRAS+HES APC (AUC: 0.892, 95% CI 0.885 to 0.899) (table 3). Calibration slopes and intercepts were close to 1 and 0, respectively, using all individual and combined data sources. There was little variation in the calibration metrics when using the different data sources to define cancer (table 3). Similar patterns in model performance were observed across datasets when measuring OC incidence within 6 and 18 months (online supplemental Table 3).

**Table 3:** Ovatools model performance by data source

Model	AUC (95% CI)	Calibration intercept (95% CI)	Calibration slope (95% CI)	O:E	CITL
NCRAS	0.924 (0.917 to 0.931)	−0.001 (−0.051 to 0.049)	1.019 (0.773 to 1.265)	0.887	−0.161
CPRD	0.903 (0.895 to 0.910)	−0.001 (−0.049 to 0.048)	0.940 (0.712 to 1.168)	0.875	−0.180
HES APC	0.920 (0.913 to 0.927)	0.951 (0.734 to 1.168)	1.032 (0.774 to 1.290)	0.985	−0.020
CPRD+NCRAS	0.902 (0.895 to 0.908)	0.001 (−0.042 to 0.045)	0.987 (0.725 to 1.249)	1.136	0.171
CPRD+NCRAS+HES APC	0.892 (0.885 to 0.899)	0.003 (−0.038 to 0.043)	0.977 (0.702 to 1.252)	1.298	0.352

AUC, area under curve; CITL, calibration-in-the-large; CPRD, Clinical Practice Research Datalink; HES APC, Hospital Episode Statistics Admitted Patient Care; NCRAS, National Cancer Registration and Analysis Service; O:E, observed versus expected.

### Diagnostic accuracy

Using the ≥3% risk threshold, sensitivity was highest when using individual data sources: NCRAS (73.2%; 95% CI 71.4% to 74.9%), HES APC (72.9%; 95% CI 71.2% to 74.5%) and CPRD (68.8%; 95% CI 66.9% to 70.6%), while sensitivity was lower when combining data sources: CPRD+NCRAS (67.8%; 95% CI 66.1% to 69.4%) and CPRD+NCRAS+HES APC (65.4%; 95% CI 63.8% to 66.9%) (table 4). PPV was lowest using individual data sources: with CPRD having the lowest PPV (13.8%; 95% CI 13.2% to 14.4%), with slight increases for NCRAS (14.8%; 95% CI 14.2% to 15.5%) and HES APC (16.4%; 95% CI 15.8% to 17.1%). PPV was markedly higher using combined data sources CPRD+NCRAS (17.6%; 95% CI 16.9% to 18.3%) and CPRD+NCRAS+HES APC (19.4%; 95% CI 18.7% to 20.1%) (table 4). Similar patterns were observed using a ≥1% risk threshold and using the current CA125 cut-off (≥35 U/mL) (online supplemental Table 4). Similar patterns in sensitivity and PPV were observed across data sources when using OC incidence at 6 and 18 months (online supplemental Table 5). To support interpretation, a full 2×2 contingency table for each data source for OC within 12 months of index CA125 at ≥3% risk was included (online supplemental Table 6).

**Table 4:** Diagnostic accuracy of Ovatools at >3% risk to detect OC within 12 months of index CA125 by data source

Data source (12 month OC incidence)	Sensitivity, % (95% CI)	Specificity, % (95% CI)	PPV, % (95% CI)	NPV, % (95% CI)
NCRAS (0.75%)	73.2 (71.4 to 74.9)	96.8 (96.8 to 96.9)	14.8 (14.2 to 15.5)	99.8 (99.8 to 99.8)
HES APC (0.84%)	72.9 (71.2 to 74.5)	96.9 (96.8 to 96.9)	16.4 (15.8 to 17.1)	99.8 (99.7 to 99.8)
CPRD (0.74%)	68.8 (66.9 to 70.6)	96.8 (96.7 to 96.8)	13.8 (13.2 to 14.4)	99.8 (99.7 to 99.8)
CPRD+NCRAS (0.97%)	67.8 (66.1 to 69.4)	96.9 (96.8 to 97.0)	17.6 (16.9 to 18.3)	99.7 (99.7 to 99.7)
CPRD+NCRAS+HES APC (1.10%)	65.4 (63.8 to 66.9)	97.0 (96.9 to 97.0)	19.4 (18.7 to 20.1)	99.6 (99.6 to 99.6)

CA125, cancer antigen; CPRD, Clinical Practice Research Datalink; HES APC, Hospital Episode Statistics Admitted Patient Care; NCRAS, National Cancer Registration and Analysis Service; NPV, negative predictive value; OC, ovarian cancer; PPV, positive predictive value.

### Code-level concordance between CPRD, HES APC and NCRAS

As a secondary analysis, we examined the concordance between CPRD and HES APC diagnostic codes with NCRAS. Comparing CPRD and NCRAS, CPRD codes explicitly denoting malignant OC demonstrated high concordance with NCRAS. In contrast, borderline or non-specific OC diagnostic codes were less frequently confirmed in NCRAS (online supplemental Table 7). Similar patterns were observed between HES APC and NCRAS, with malignant ICD-10 codes demonstrating higher agreement and borderline showing lower confirmation rates (online supplemental Table 8).

## Discussion

### Summary of findings

This study measured the variation in diagnostic accuracy of an OC prediction model when different data sources were used to define OC diagnosis. Our findings demonstrate that while model calibration was similar, all other accuracy metrics varied with the source of outcome data used. AUC was highest when using NCRAS data alone. At the ≥3% risk threshold, the model’s sensitivity was highest using NCRAS data alone (73.2%), closely followed by HES APC (72.9%), with lower sensitivity when using CPRD and combined data sources. PPV was markedly higher when using combined data sources CPRD+NCRAS+HES APC (19.4%) and CPRD+NCRAS (17.6%) compared with NCRAS data alone (14.8%). The consistent performance patterns across alternative thresholds and follow-up windows suggest these differences were unlikely to be driven by the model and instead reflect heterogeneity in OC outcomes across data sources.

### Strengths and limitations

To our knowledge, this is the first study to examine the impact on prediction model performance and threshold accuracy when using different routinely collected data sources to define cancer. Our study leveraged three widely used and nationally representative datasets, providing a comprehensive analysis of OC records across English primary care, secondary care and CR. The inclusion of combined data sources to define incident OC allowed for a detailed investigation into cancer incidence. Variation in incidence and accuracy metrics can occur for CPRD and HES depending on codes selected, as codes can be non-specific. Codes to define OC (ICD-10, SNOMED CT) were checked by two clinicians (KDDA and GF) to ensure appropriate code inclusion. While the cohort for Ovatools model development used a different data source (CPRD GOLD) to that used to define the cohort in this study (CPRD Aurum), NCRAS data was used to define the OC outcome in both studies, so model performance (calibration and to a lesser extent discrimination) may be expected to be better when using NCRAS data as the outcome rather than CPRD or HES in the current study. Although the study cohort excluded patients with a history of OC uniformly across all analysis to ensure cohort consistency, the number of excluded cases from each data source was small and therefore unlikely to have influenced the study findings or conclusions.

We studied a single and relatively simple model for one cancer type. Accuracy and completeness of records between data sources may vary by cancer type. Moreover, we used HES APC as our only secondary care dataset. Other HES datasets, such as HES Accident & Emergency or HES outpatient, may contain OC records which were not included in this study. As a result, OC records only recorded in non-HES APC datasets may have been missed. Consequently, our findings may not be generalisable to other models and cancers.^27^ Additionally, model performance was evaluated at a single risk threshold of 3%, chosen to align with NICE NG12 guidance and existing primary care cancer prediction literature. However, alternative thresholds may result in various trade-offs between sensitivity and specificity, which were not explored in this study.^28^

### Comparison with existing literature

Several studies have examined the differences between English cancer records in CR and non-CR data sources, and the potential impact this has for research and practice.^13^,^15^ Previous research shows that when compared with using the gold standard for cancer records (NCRAS), HES data had a greater concordance than CPRD data.^15 29^ While we did not evaluate concordance in our study, we noted a higher OC incidence in HES APC than CPRD. The lower incidence and concordance in CPRD compared with NCRAS may be explained by the delay between cancer diagnosis in secondary care and follow-up in primary care or receipt of discharge letters, possibly weeks or months after diagnosis. Furthermore, CPRD relies on primary care clinicians correctly coding cancer diagnoses at the correct time or back-dating records for accurate timing.^4^ In comparison, NCRAS records involve robust quality control and assurance checks where each cancer record is rigorously checked to address inconsistencies prior to the finalisation of records.^12^ Dregan *et al*^30^ demonstrated in the General Practice Research Database, an older version of CPRD, that most cancer codes were recorded 11 days later compared with NCRAS, which was similarly shown by Arhi *et al.*^13^ This introduces potential errors such as miscoding, misclassification of suspected cancers or missed diagnosis.

HES APC is an admitted patient secondary care database comprising records made during inpatient episodes, and so does not capture records made in outpatient clinics. As cancers are usually diagnosed in secondary care, cancer records may be better recorded in secondary care data sources than in primary care data sources (eg, CPRD), as observed in Strongman *et al*. and Somathilake *et al*’s studies.^15 29^ However, we measured a higher OC incidence in HES APC than NCRAS, which could be explained either by recording errors in hospital data or by the lack of ability to determine ovarian tumour behaviour (invasive, borderline or benign) in HES APC, and thus some benign tumours may potentially be misclassified as cancer. Being unable to differentiate tumour behaviour in non-CR sources is even more challenging in CPRD, where coding systems are not standardised and change frequently. Invasive OCs are more aggressive than borderline ovarian tumours, and thus it is essential to be able to differentiate this in the outcome when developing and evaluating predictive models.

The potential misclassification of cancer records in HES APC and CPRD, and delay in recording of data in CPRD could explain variation in model performance and accuracy. For example, the lower sensitivity when using CPRD alone or in combination could result from miscoding of patients without cancer, for example, benign disease or suspected cancer (misclassification bias), while the higher PPV when using multiple data sources is expected as disease incidence (including miscoded patients) is higher.

### Implications for research and practice

While some prediction model studies have used NCRAS to define cancer, many have relied on non-CR data sources.^16 17^ This may be due to practical barriers, such as linking between datasets such as CPRD and NCRAS, introducing significant administrative and logistical barriers, often extending project timelines by 3–6 months and incurring substantial costs.^11^ Moreover, NCRAS data is only available several years after collection due to the extensive time needed to collate information and produce validated data releases.^12^ As such, the trade-off between cost, time and accuracy may lead researchers to use non-CR data sources.

Some studies have used combinations of NCRAS and non-CR data to define cancer cases.^6^ In our study, using multiple sources of outcome data led to an increase in cancer incidence, modest reductions in discrimination and sensitivity and an increase in PPV when compared with using single sources of outcome data. While a proportion of these additional cases had an abnormal Ovatools result or CA125 level, a significant fraction of the additional cases had normal test results (false negatives), resulting in lower sensitivity and AUC. In our secondary analysis, non-CR codes denoting borderline disease and non-specific diagnostic codes showed lower concordance. However, even for the most common CPRD code, which is specific for malignancy (malignant neoplasm of the ovary), 16.6% of cases were not confirmed in NCRAS, so using more specific CPRD codes would not fully resolve the differences in accuracy observed.

Our findings indicate that using multiple data sources overestimates cancer incidence (likely through the inclusion of some people who do not have cancer), leading to an invalid assessment of performance and accuracy. When developing and validating models, this could result in researchers selecting lower, suboptimal, model action thresholds to ensure sufficient sensitivity which would lead to more unnecessary referrals and harm if the model entered clinical practice. Inaccurate estimates of performance also hinder informed decision making by clinicians and doctors. For example, a patient may choose not to undergo a test if its ability to pick up cancer is limited, while an apparent higher PPV (often used to help interpret test results) could result in unnecessary further investigations and referrals as perceived risk is higher.

Future research is needed to investigate the impact of using different data sources for predictive modelling for other cancer types within the UK, particularly in primary care where early detection tools are often deployed. The NHS Long Term Plan target aims for 75% of cancers to be diagnosed at an early stage by 2028.^31^ With the limited availability of cost-effective screening programmes, particularly for rare cancers, early diagnostic strategies in primary care are essential to work toward this target. Cancer predictive models have the potential to be widely used to improve timely cancer diagnosis in English primary care settings, but to maximise their potential effectiveness in real-world settings, it is important to use the most accurate and reliable sources of cancer records to develop and evaluate the models.

## Supplementary material

10.1136/bmjph-2025-004229 online supplemental file 1

## Data Availability

Data may be obtained from a third party and are not publicly available.
