# Supplementary material for: Impact of cancer outcome data source on the diagnostic accuracy of ovarian cancer prediction models: a primary care cohort study
Source: BMJ Public Health. 2026 Mar 25;4(1):e004229. doi: 10.1136/bmjph-2025-004229 (PMC13034229; doi:10.1136/bmjph-2025-004229)
Supplement: online supplemental file 1 [file bmjph-4-1-s001.docx]

Table S1: CPRD code list for ovarian cancer with SNOMED CT Concept ID, medcodeid and term

| **SNOMED CT Concept ID** | **medcodeid** | **Term** |
| --- | --- | --- |
| 363421004 | 6245501000006114 | ca - cancer of omentum |
| 363421004 | 6245491000006118 | cancer of omentum |
| 449377002 | 7369071000006110 | cancer of pelvic peritoneum |
| 363370001 | 482537012 | malignant neoplasm of mesentery |
| 363421004 | 482641017 | malignant neoplasm of omentum |
| 93947009 | 155446017 | malignant neoplasm of parietal peritoneum |
| 449377002 | 155452016 | malignant neoplasm of pelvic peritoneum |
| 187814001 | 288754013 | malignant neoplasm of the pouch of douglas |
| 93947009 | 4027261000006117 | primary malignant neoplasm of parietal peritoneum |
| 363492001 | 292132011 | [x]malignant neoplasm of peritoneum, unspecified |
| 363492001 | 6247371000006118 | ca - cancer of peritoneum |
| 363492001 | 6247381000006115 | cancer of peritoneum |
| 187808008 | 288747019 | malignant neoplasm of specified parts of peritoneum |
| 187808008 | 288756010 | malignant neoplasm of specified parts of peritoneum nos |
| 363492001 | 6247351000006111 | malignant tumor of peritoneum |
| 363492001 | 6247341000006114 | malignant tumour of peritoneum |
| 363492001 | 6247361000006113 | peritoneal cancer |
| 15674004 | 2748631000006115 | primary serous papillary carcinoma of peritoneum |
| 67182003 | 407801000000116 | [m]borderline mucinous cystadenoma of the ovary |
| 42194009 | 306091000006115 | [m]brenner tumour, malignant |
| 18105004 | 307921000006115 | [m]endometrioid adenofibroma, malignant |
| 18861007 | 309551000006112 | [m]granulosa cell tumour, malignant |
| 79143006 | 291515019 | [m]mucinous cystadenocarcinoma nos |
| 67182003 | 1232889015 | [m]mucinous cystadenoma nos |
| 128852007 | 313111000006119 | [m]mucinous cystadenoma, borderline malignancy |
| 2735009 | 291510012 | [m]papillary cystadenocarcinoma, nos |
| 128850004 | 314851000006110 | [m]papillary cystadenoma, borderline malignancy |
| 128850004 | 1220825014 | [m]papillary cystadenoma, borderline malignancy |
| 68880006 | 314921000006110 | [m]papillary mucinous cystadenocarcinoma |
| 90282004 | 314971000006111 | [m]papillary serous cystadenocarcinoma |
| 128851000 | 1220826010 | [m]papillary serous cystadenoma, borderline malignancy |
| 128851000 | 314991000006112 | [m]papillary serous cystadenoma, borderline malignancy |
| 79143006 | 315741000006117 | [m]pseudomucinous adenocarcinoma |
| 67182003 | 315751000006115 | [m]pseudomucinous cystadenoma nos |
| 90725004 | 291508010 | [m]serous cystadenocarcinoma, nos |
| 128849004 | 316381000006117 | [m]serous cystadenoma, borderline malignancy |
| 128849004 | 1220823019 | [m]serous cystadenoma, borderline malignancy |
| 15674004 | 316401000006117 | [m]serous surface papillary carcinoma |
| 18854008 | 317041000006111 | [m]struma ovarii, malignant |
| 907041000006102 | 907041000006118 | [rfc] cancer of the ovary |
| 128849004 | 4418141000006113 | atypical proliferating serous tumor |
| 128849004 | 4418151000006110 | atypical proliferating serous tumour |
| 128852007 | 4418341000006111 | atypical proliferative mucinous tumor |
| 128852007 | 4418361000006110 | atypical proliferative mucinous tumour |
| 128851000 | 4418261000006117 | atypical proliferative papillary serous tumor |
| 128851000 | 4418291000006113 | atypical proliferative papillary serous tumour |
| 42194009 | 3180121000006115 | brenner tumor, malignant |
| 42194009 | 3180111000006111 | brenner tumour, malignant |
| 363443007 | 6246131000006116 | ca - cancer of ovary |
| 363443007 | 1228570010 | cancer of ovary |
| 18105004 | 2787491000006111 | endometrioid adenofibroma, malignant |
| 30289006 | 2987761000006118 | endometrioid cystadenocarcinoma |
| 18105004 | 2787501000006115 | endometrioid cystadenofibroma, malignant |
| 18861007 | 2799411000006113 | granulosa cell carcinoma |
| 18861007 | 2799401000006110 | granulosa cell tumor, malignant |
| 18861007 | 2799421000006117 | granulosa cell tumor, sarcomatoid |
| 18861007 | 2799391000006113 | granulosa cell tumour, malignant |
| 18861007 | 2799431000006119 | granulosa cell tumour, sarcomatoid |
| 42194009 | 3180141000006110 | malignant brenner tumor |
| 42194009 | 3180131000006117 | malignant brenner tumour |
| 18105004 | 2787521000006113 | malignant endometrioid adenofibroma |
| 18105004 | 2787511000006117 | malignant endometrioid cystadenofibroma |
| 18861007 | 2799451000006114 | malignant granulosa cell tumor |
| 363443007 | 723281000006111 | malignant neoplasm of ovary |
| 18854008 | 2799271000006115 | malignant struma ovarii |
| 363443007 | 6246121000006119 | malignant tumor of ovary |
| 90282004 | 3967621000006113 | micropapillary serous carcinoma |
| 128852007 | 4418411000006119 | mucinous borderline tumor |
| 128852007 | 4418401000006117 | mucinous borderline tumour |
| 128852007 | 4418331000006118 | mucinous cystadenoma, borderline malignancy |
| 128852007 | 4418351000006113 | mucinous cystic tumor of borderline malignancy |
| 128852007 | 4418301000006114 | mucinous cystic tumour of borderline malignancy |
| 128852007 | 4418321000006116 | mucinous tumor of low malignant potential |
| 128852007 | 4418371000006115 | mucinous tumour of low malignant potential |
| 363443007 | 6246151000006111 | ovarian cancer |
| 128850004 | 4418221000006111 | papillary cystadenoma, borderline malignancy |
| 68880006 | 3620201000006112 | papillary mucinous cystadenocarcinoma |
| 68880006 | 3620221000006119 | papillary pseudomucinous adenocarcinoma |
| 68880006 | 3620211000006110 | papillary pseudomucinous cystadenocarcinoma |
| 90282004 | 3967611000006117 | papillary serous adenocarcinoma |
| 90282004 | 3967601000006115 | papillary serous cystadenocarcinoma |
| 128851000 | 4418251000006119 | papillary serous cystadenoma, borderline malignancy |
| 128851000 | 4418241000006116 | papillary serous tumor of low malignant potential |
| 128851000 | 4418281000006110 | papillary serous tumour of low malignant potential |
| 2735009 | 2542411000006113 | papillocystic adenocarcinoma |
| 79143006 | 3787041000006119 | pseudomucinous adenocarcinoma |
| 79143006 | 3787051000006117 | pseudomucinous cystadenocarcinoma |
| 128852007 | 4418381000006117 | pseudomucinous cystadenoma - borderline malignancy |
| 128852007 | 4418311000006112 | pseudomucinous cystadenoma, borderline malignancy |
| 90725004 | 3974511000006118 | serous adenocarcinoma |
| 128849004 | 4418181000006119 | serous borderline tumor |
| 128849004 | 4418201000006118 | serous borderline tumour |
| 90725004 | 3974521000006114 | serous carcinoma |
| 128849004 | 4418121000006118 | serous cystadenoma, borderline malignancy |
| 128851000 | 4418271000006112 | serous papillary cystic tumor of borderline malignancy |
| 128851000 | 4418231000006114 | serous papillary cystic tumour of borderline malignancy |
| 128849004 | 4418131000006115 | serous tumor of low malignant potential |
| 128849004 | 4418191000006116 | serous tumor, atypical proliferative |
| 128849004 | 4418161000006112 | serous tumour of low malignant potential |
| 128849004 | 4418211000006115 | serous tumour, atypical proliferative |
| 18854008 | 2799261000006110 | struma ovarii, malignant |
| 533411000000108 | 1222189010 | ca ovary/other uterine adnexa |
| 573671000000104 | 880411000006116 | ca ovary/uterine adnexa nos |
| 428322007 | 289199018 | malignant neoplasm of ovary and other uterine adnexa |
| 533411000000108 | 12718971000006119 | malignant neoplasm of ovary and other uterine adnexa |
| 363444001 | 726271000006117 | malignant neoplasm of fallopian tube |
| 363444001 | 6246171000006118 | malignant tumor of fallopian tube |
| 363444001 | 6246161000006113 | malignant tumour of fallopian tube |
| 62283005 | 291571017 | [m]androblastoma nos |
| 62283005 | 305631000006116 | [m]arrhenoblastoma nos |
| 89996007 | 306081000006118 | [m]brenner tumour, borderline malignancy |
| 2962009 | 307321000006116 | [m]cystadenofibroma nos |
| 115217007 | 291492015 | [m]cystic, mucinous and serous neoplasms |
| 115217007 | 291529018 | [m]cystic, mucinous or serous neoplasm nos |
| 20829008 | 291464013 | [m]endometrioid adenofibroma nos |
| 25874003 | 307911000006111 | [m]endometrioid adenofibroma, borderline malignancy |
| 123844007 | 291465014 | [m]endometrioid adenoma or carcinoma nos |
| 75987005 | 307951000006112 | [m]endometrioid adenoma, borderline malignancy |
| 123844007 | 291462012 | [m]endometrioid adenomas and carcinomas |
| 74751003 | 309451000006111 | [m]gonadoblastoma |
| 417629008 | 291566012 | [m]granulosa cell tumour nos |
| 31296004 | 309561000006114 | [m]granulosa cell-theca cell tumour |
| 26735007 | 309581000006116 | [m]gynandroblastoma |
| 77029009 | 1216937017 | [m]juvenile granulosa cell tumour |
| 115217007 | 403753016 | [m]ovarian cystic, mucinous and serous neoplasms |
| 115217007 | 291524011 | [m]ovarian cystic, mucinous or serous neoplasm nos |
| 115217007 | 314581000006117 | [m]ovarian mucinous tumour |
| 115217007 | 376842018 | [m]ovarian papillary tumour |
| 417629008 | 376849010 | [m]ovarian stromal tumour |
| 72457004 | 1216896013 | [m]sex cord tumour with annular tubules |
| 115221000 | 291578011 | [m]specialised gonadal neoplasm nos |
| 115221000 | 291553012 | [m]specialised gonadal neoplasms |
| 32071008 | 317051000006113 | [m]strumal carcinoid |
| 32071008 | 291728011 | [m]strumal neoplasms |
| 62283005 | 3511981000006117 | androblastoma |
| 62283005 | 3512001000006117 | androblastoma, no icd-o subtype |
| 62283005 | 3512011000006119 | androblastoma, no international classification of diseases for oncology subtype |
| 62283005 | 3511991000006119 | arrhenoblastoma |
| 75987005 | 3735801000006114 | atypical proliferative endometrioid tumor |
| 75987005 | 3735811000006112 | atypical proliferative endometrioid tumour |
| 89996007 | 3963051000006115 | borderline brenner tumor |
| 89996007 | 3963031000006110 | borderline brenner tumour |
| 89996007 | 3963021000006112 | brenner tumor - borderline malignancy |
| 89996007 | 3963061000006118 | brenner tumor, atypical proliferative |
| 89996007 | 3962961000006111 | brenner tumor, borderline malignancy |
| 89996007 | 3962971000006116 | brenner tumor, proliferating |
| 89996007 | 3962991000006115 | brenner tumour - borderline malignancy |
| 89996007 | 3963041000006117 | brenner tumour, atypical proliferative |
| 89996007 | 3962951000006114 | brenner tumour, borderline malignancy |
| 89996007 | 3962981000006118 | brenner tumour, proliferating |
| 25874003 | 2914101000006114 | endometrioid adenofibroma - borderline malignancy |
| 25874003 | 2914071000006116 | endometrioid adenofibroma, borderline malignancy |
| 75987005 | 3735821000006116 | endometrioid adenoma - borderline malignancy |
| 75987005 | 3735761000006117 | endometrioid adenoma, borderline malignancy |
| 20829008 | 2831281000006114 | endometrioid cystadenofibroma |
| 25874003 | 2914091000006115 | endometrioid cystadenofibroma - borderline malignancy |
| 25874003 | 2914081000006118 | endometrioid cystadenofibroma, borderline malignancy |
| 75987005 | 3735831000006118 | endometrioid cystadenoma - borderline malignancy |
| 75987005 | 3735771000006112 | endometrioid cystadenoma, borderline malignancy |
| 128726006 | 4414031000006113 | endometrioid stromal sarcoma, low grade |
| 75987005 | 3735781000006110 | endometrioid tumor of low malignant potential |
| 75987005 | 3735851000006113 | endometrioid tumor, borderline |
| 75987005 | 3735791000006113 | endometrioid tumour of low malignant potential |
| 75987005 | 3735841000006111 | endometrioid tumour, borderline |
| 74751003 | 3714981000006118 | gonocytoma |
| 31296004 | 3004541000006116 | granulosa cell - theca cell tumor |
| 31296004 | 3004521000006111 | granulosa cell - theca cell tumour |
| 31296004 | 3004481000006111 | granulosa cell-theca cell tumor |
| 31296004 | 3004471000006113 | granulosa cell-theca cell tumour |
| 26735007 | 2928221000006110 | gynandroblastoma |
| 77029009 | 3752601000006117 | juvenile granulosa cell tumor |
| 77029009 | 3752591000006113 | juvenile granulosa cell tumour |
| 26372004 | 2922171000006119 | luteal cell tumour |
| 94974001 | 4048251000006116 | neoplasm of uncertain behavior of ovary |
| 94974001 | 511728010 | neoplasm of uncertain behaviour of ovary |
| 71440001 | 3662171000006118 | ovarian stromal tumor |
| 71440001 | 3662191000006117 | ovarian stromal tumour |
| 89996007 | 3963011000006116 | proliferating brenner tumor |
| 89996007 | 3963001000006119 | proliferating brenner tumour |
| 128694007 | 11908881000006116 | serous surface papillary tumor of borderline malignancy |
| 128694007 | 4413191000006111 | serous surface papillary tumour of borderline malignancy |
| 72457004 | 3678241000006112 | sex cord tumor with annular tubules |
| 72457004 | 3678231000006119 | sex cord tumour with annular tubules |
| 417629008 | 6909731000006119 | sex cord-stromal tumour category |
| 115221000 | 4255491000006110 | specialised gonadal neoplasm |
| 115221000 | 11989581000006111 | specialized gonadal neoplasm |
| 115221000 | 11908991000006114 | specialized gonadal neoplasm |
| 32071008 | 3016461000006114 | struma ovarii and carcinoid |
| 32071008 | 3016451000006112 | strumal carcinoid |
| 768795009 | 12079021000006115 | surface epithelial-stromal tumour |
| 31296004 | 3004531000006114 | theca cell - granulosa cell tumor |
| 31296004 | 3004511000006115 | theca cell - granulosa cell tumour |
| 31296004 | 3004491000006114 | theca cell-granulosa cell tumor |
| 31296004 | 3004501000006118 | theca cell-granulosa cell tumour |

Table S2: Ovarian cancer incidence within 6, 12 and 18 months of index CA125, using different data sources to define the outcome

| **Data source** | **Incidence period after index CA125** | **Number (n)** | **Proportion (%)** |
| --- | --- | --- | --- |
| **NCRAS** | 6 months | 2,453 | 0.72 |
|  | 12 months | 2,571 | 0.75 |
|  | 18 months | 2,634 | 0.77 |
| **CPRD** | 6 months | 2,332 | 0.68 |
|  | 12 months | 2,535 | 0.74 |
|  | 18 months | 2,642 | 0.77 |
| **HES APC** | 6 months | 2,632 | 0.77 |
|  | 12 months | 2,855 | 0.84 |
|  | 18 months | 2,975 | 0.87 |
| **CPRD + NCRAS** | 6 months | 3,080 | 0.90 |
|  | 12 months | 3,291 | 0.97 |
|  | 18 months | 3,400 | 1.00 |
| **CPRD + NCRAS + HES APC** | 6 months | 3,475 | 1.02 |
|  | 12 months | 3,761 | 1.10 |
|  | 18 months | 3,913 | 1.14 |
| **CPRD** - Clinical Practice Research Datalink, **HES APC** - Hospital Episode Statistics, **OC** – Ovarian cancer, **NCRAS** - National Cancer Registration and Analysis Service | | | |

Table S3: Model performance metrics of Ovatools to detect ovarian cancer within 6 and 18 months, by data source

| **6 months** | | | | | |
| --- | --- | --- | --- | --- | --- |
| **Data source** | **AUC (95% CI)** | **Calibration intercept (95% CI)** | **Calibration slope (95% CI)** | **O:E** | **CITL** |
| **NCRAS** | 0.934  (0.928, 0.941) | -0.002  (-0.054, 0.050) | 1.048  (0.800, 1.296) | 0.846 | -0.224 |
| **CPRD** | 0.919  (0.911, 0.926) | -0.001  (-0.053, 0.050) | 0.977  (0.747, 1.207) | 0.805 | -0.292 |
| **HES APC** | 0.933  (0.927, 0.940) | -0.001  (-0.052, 0.049) | 1.068  (0.810, 1.326) | 0.908 | -0.129 |
| **CPRD + NCRAS** | 0.916  (0.910, 0.923) | 0.000  (-0.045, 0.046) | 1.026  (0.762, 1.290) | 1.063 | 0.082 |
| **CPRD + NCRAS + HES APC** | 0.907  (0.901, 0.914) | 0.002  (-0.041, 0.044) | 1.019  (0.742, 1.296) | 1.199 | 0.245 |
| **95% CI** - Confidence interval, **AUC** – Area Under Curve, **CPRD** - Clinical Practice Research Datalink, **HES APC** - Hospital Episode Statistics, **O:E** – Observed versus expected, **NCRAS** - National Cancer Registration and Analysis Service | | | | | |

| **18 months** | | | | | |
| --- | --- | --- | --- | --- | --- |
| **Data source** | **AUC (95% CI)** | **Calibration intercept (95% CI)** | **Calibration slope (95% CI)** | **O:E** | **CITL** |
| **NCRAS** | 0.919  (0.911, 0.926) | -0.078  (-0.127, -0.029) | 1.005  (0.760, 1.125) | 0.909 | -0.128 |
| **CPRD** | 0.894  (0.886, 0.901) | 0.000  (-0.047, 0.047) | 0.922  (0.694, 1.150) | 0.912 | -0.124 |
| **HES APC** | 0.911  (0.904, 0.918) | 0.000  (-0.046, 0.046) | 1.011  (0.753, 1.269) | 1.026 | 0.035 |
| **CPRD + NCRAS** | 0.894  (0.887, 0.901) | 0.002  (-0.041, 0.044) | 0.967  (0.706, 1.228) | 1.173 | 0.215 |
| **CPRD + NCRAS + HES APC** | 0.883  (0.876, 0.890) | 0.003  (-0.036, 0.043) | 0.953  (0.680, 1.226) | 1.35 | 0.405 |
| **95% CI** - Confidence interval, **AUC** – Area Under Curve, **CPRD** - Clinical Practice Research Datalink, **HES APC** - Hospital Episode Statistics, **O:E** – Observed versus expected**, NCRAS** - National Cancer Registration and Analysis Service | | | | | |

Table S4: Diagnostic accuracy of Ovatools and CA125>35U/ml to detect any ovarian cancer at 12 months of CA125

| **Data source (OC incidence)** | **CA125 or Ovatools threshold** | **Sensitivity, % (95% CI)** | **Specificity, % (95% CI)** | **PPV, % (95% CI)** | **NPV, % (95% CI)** |
| --- | --- | --- | --- | --- | --- |
| **CPRD (0.74%)** | CA125 >35U/mL | 72.7 (71.0, 74.5) | 93.6 (93.5, 93.7) | 7.9 (7.5, 8.2) | 99.8 (99.8, 99.8) |
|  | >1% | 80.3 (78.7, 81.8) | 88.0 (87.9, 88.1) | 4.8 (4.6, 5.0) | 99.8 (99.8, 99.8) |
|  | >3% | 68.8 (66.9, 70.6) | 96.8 (96.7, 96.8) | 13.8 (13.2, 14.4) | 99.8 (99.7, 99.8) |
| **NCRAS (0.75%)** | CA125 >35U/mL | 78.3 (76.7, 79.9) | 93.7 (93.6, 93.8) | 8.6 (8.2, 9.0) | 99.8 (99.8, 99.8) |
|  | >1% | 85.2 (83.8, 86.6) | 88.0 (87.9, 88.1) | 5.1 (4.9, 5.3) | 99.9 (99.9, 99.9) |
|  | >3% | 73.2 (71.4, 74.9) | 96.8 (96.8, 96.9) | 14.8 (14.2, 15.5) | 99.8 (99.8, 99.8) |
| **HES (0.84%)** | CA125 >35U/mL | 77.7 (76.1, 79.2) | 93.7 (93.6, 93.8) | 9.5 (9.1, 9.9) | 99.8 (99.8, 99.8) |
|  | >1% | 84.3 (83.0, 85.7) | 88.1 (88.0, 88.2) | 5.6 (5.4, 5.9) | 99.9 (99.8, 99.9) |
|  | >3% | 72.9 (71.2, 74.5) | 96.9 (96.8, 96.9) | 16.4 (15.8, 17.1) | 99.8 (99.7, 99.8) |
| **CPRD + NCRAS (0.97%)** | CA125 >35U/mL | 72.7 (71.1, 74.2) | 93.8 (93.7, 93.9) | 10.2 (9.8, 10.6) | 99.7 (99.7, 99.7) |
|  | >1% | 80.1 (78.7, 81.5) | 88.1 (88.0, 88.2) | 6.2 (5.9, 6.4) | 99.8 (99.8, 99.8) |
|  | >3% | 67.8 (66.1, 69.4) | 96.9 (96.8, 97.0) | 17.6 (16.9, 18.3) | 99.7 (99.7, 99.7) |
| **CPRD + NCRAS + HES (1.10%)** | CA125 >35U/mL | 70.6 (69.1, 72.1) | 93.8 (93.8, 93.9) | 11.3 (10.9, 11.8) | 99.7 (99.6, 99.7) |
|  | >1% | 78.3 (77.0, 79.6) | 88.2 (88.1, 88.3) | 6.9 (6.7, 7.1) | 99.7 (99.7, 99.7) |
|  | >3% | 65.4 (63.8, 66.9) | 97.0 (96.9, 97.0) | 19.4 (18.7, 20.1) | 99.6 (99.6, 99.6) |
| **95% CI** - Confidence interval, **CA125** - Cancer antigen 125, **CPRD** - Clinical Practice Research Datalink, **HES APC** - Hospital Episode Statistics, **NCRAS** - National Cancer Registration and Analysis Service, **NPV** - Negative predictive value, **OC** - Ovarian cancer, **PPV** - Positive predictive value | | | | | |

Table S5: Diagnostic accuracy of Ovatools and CA125 >35U/ml to detect any ovarian cancer within 6 and 18 months of index CA125

| **Ovarian cancer at 6 months** | | | | | |
| --- | --- | --- | --- | --- | --- |
| **Data source**  **(6-month OC incidence)** | **CA125 or Ovatools threshold** | **Sensitivity, % (95% CI)** | **Specificity, % (95% CI)** | **PPV, %**  **(95% CI)** | **NPV, %**  **(95% CI)** |
| **CPRD (0.69%)** | CA125 >35U/mL | 76.5  (74.7, 78.2) | 93.6  (93.5, 93.7) | 7.6  (7.3, 8.0) | 99.8  (99.8, 99.8) |
|  | >1% | 83.5  (81.9, 85.0) | 87.9  (87.8, 88.1) | 4.6  (4.4, 4.8) | 99.9  (99.9, 99.9) |
|  | >3% | 72.6  (70.7, 74.4) | 96.8  (96.7, 96.8) | 13.4  (12.8, 14.0) | 99.8  (99.8, 99.8) |
| **NCRAS (0.72%)** | CA125 >35U/mL | 80.8  (79.2, 82.4) | 93.7  (93.6, 93.7) | 8.5  (8.1, 8.8) | 99.9  (99.8, 99.9) |
|  | >1% | 87.3  (85.9, 88.6) | 88.0  (87.9, 88.1) | 5.0  (4.8, 5.2) | 99.9  (99.9, 99.9) |
|  | >3% | 75.7  (74.0, 77.4) | 96.8  (96.7, 96.9) | 14.7  (14.0, 15.3) | 99.8  (99.8, 99.8) |
| **HES (0.77%)** | CA125 >35U/mL | 81.2  (79.6, 82.6) | 81.2  (79.6, 82.6) | 9.1  (8.8, 9.5) | 99.8  (99.8, 99.9) |
|  | >1% | 87.3  (86.0, 88.6) | 88.0  (87.9, 88.1) | 5.4  (5.2, 5.6) | 99.9  (99.9, 99.9) |
|  | >3% | 76.6  (74.9, 78.2) | 96.8  (96.8, 96.9) | 15.9  (15.3, 16.6) | 99.8  (99.8, 99.8) |
| **CPRD + NCRAS (0.91%)** | CA125 >35U/mL | 76.0  (74.5, 77.5) | 93.8  (93.7, 93.8) | 10.0  (9.6, 10.4) | 99.8  (99.7, 99.8) |
|  | >1% | 83.1  (81.7, 84.4) | 88.1  (88.0, 88.2) | 6.0  (5.8, 6.2) | 99.8  (99.8, 99.8) |
|  | >3% | 71.1  (69.5, 72.7) | 96.9  (96.8, 97.0) | 17.3  (16.6, 18.0) | 99.7  (99.7, 99.7) |
| **CPRD + NCRAS + HES (1.02%)** | CA125 >35U/mL | 74.4  (72.9, 75.8) | 93.8  (93.7, 93.9) | 11.0  (10.6, 11.4) | 99.7  (99.7, 99.7) |
|  | >1% | 81.6  (80.3, 82.9) | 88.2  (88.1, 88.3) | 6.6 (6.4, 6.9) | 99.8  (99.8, 99.8) |
|  | >3% | 69.2  (67.6, 70.7) | 97.0  (96.9, 97.0) | 19.0  (18.3, 19.7) | 99.7  (99.7, 99.7) |
| **95% CI** - Confidence interval, **CA125** - Cancer antigen 125, **CPRD** - Clinical Practice Research Datalink, **HES APC** - Hospital Episode Statistics, **NCRAS** - National Cancer Registration and Analysis Service, **NPV** - Negative predictive value, **OC** - Ovarian cancer, **PPV** - Positive predictive value | | | | | |

| **Ovarian cancer at 18 months** | | | | | |
| --- | --- | --- | --- | --- | --- |
| **Data source**  **(18-month OC incidence)** | **CA125 or Ovatools threshold** | **Sensitivity, % (95% CI)** | **Specificity, % (95% CI)** | **PPV, %**  **(95% CI)** | **NPV, %**  **(95% CI)** |
| **CPRD (0.77%)** | CA125 >35U/mL | 70.8  (69.0, 72.5) | 93.6  (93.5, 93.7) | 8.0  (7.6, 8.3) | 99.8  (99.7, 99.8) |
|  | >1% | 78.5  (76.9, 80.1) | 88.0  (87.9, 88.1) | 4.9  (4.7, 5.1) | 99.8  (99.8, 99.8) |
|  | >3% | 66.7  (64.9, 68.5) | 96.8  (96.7, 96.8) | 13.9  (13.3, 14.5) | 99.7  (99.7, 99.7) |
| **NCRAS (0.77%)** | CA125 >35U/mL | 77.0  (75.3, 78.6) | 93.7  (93.6, 93.8) | 8.7  (8.3, 9.0) | 99.8  (99.8, 99.8) |
|  | >1% | 84.2  (82.7, 85.5) | 88.0  (87.9, 88.1) | 5.2  (5.0, 5.4) | 99.9  (99.8, 99.9) |
|  | >3% | 71.8  (70.1, 73.5) | 96.8  (96.8, 96.9) | 14.9  (14.3, 15.6) | 99.8  (99.8, 99.8) |
| **HES (0.87%)** | CA125 >35U/mL | 75.6  (74.0, 77.1) | 93.7  (93.7, 93.8) | 9.6  (9.2, 10.0) | 99.8  (99.8, 99.8) |
|  | >1% | 82.7  (81.2, 84.0) | 88.1  (88.0, 88.2) | 5.8  (5.5, 6.0) | 99.8  (99.8, 99.8) |
|  | >3% | 70.8  (69.1, 72.4) | 96.9  (96.8, 96.9) | 16.6  (16.0, 17.3) | 99.7  (99.7, 99.8) |
| **CPRD + NCRAS (1.00%)** | CA125 >35U/mL | 70.9  (69.3, 72.4) | 93.8  (93.7, 93.9) | 10.3  (9.9, 10.7) | 99.7  (99.7, 99.7) |
|  | >1% | 78.6  (77.2, 80.0) | 88.1  (88.0, 88.2) | 6.3  (6.0, 6.5) | 99.8  (99.7, 99.8) |
|  | >3% | 66.0  (64.4, 67.6) | 96.9  (96.9, 97.0) | 17.7  (17.1, 18.4) | 99.6  (99.6, 99.7) |
| **CPRD + NCRAS + HES (1.15%)** | CA125 >35U/mL | 68.5  (67.0, 70.0) | 93.8  (93.8, 93.9) | 11.4  (11.0, 11.9) | 99.6  (99.6, 99.6) |
|  | >1% | 76.5  (75.2, 77.9) | 88.2  (88.1, 88.3) | 7.0  (6.8, 7.3) | 99.7  (99.7, 99.7) |
|  | >3% | 63.3  (61.7, 64.8) | 97.0  (96.9, 97.0) | 19.5  (18.9, 20.2) | 99.6  (99.5, 99.6) |
| **95% CI** - Confidence interval, **CA125** - Cancer antigen 125, **CPRD** - Clinical Practice Research Datalink, **HES APC** - Hospital Episode Statistics, **NCRAS** - National Cancer Registration and Analysis Service, **NPV** - Negative predictive value, **OC** - Ovarian cancer, **PPV** - Positive predictive value | | | | | |

Table S6: 2×2 contingency tables for Ovatools ≥3% to detect ovarian cancer within 12 months, by data source

| **NCRAS** | | | |
| --- | --- | --- | --- |
|  | **OC within 12 months: Yes** | **OC within 12 months: No** | **Total** |
| **Ovatools ≥3%** | 1,881 (TP) | 10,786 (FP) | 12,667 |
| **Ovatools <3%** | 690 (FN) | 327,402 (TN) | 328,092 |
| **Total** | 2,571 | 338,188 | 340,759 |
| **HES APC** | | | |
|  | **OC within 12 months: Yes** | **OC within 12 months: No** | **Total** |
| **Ovatools ≥3%** | 2,080 (TP) | 10,587 (FP) | 12,667 |
| **Ovatools <3%** | 775 (FN) | 327,317 (TN) | 328,092 |
| **Total** | 2,855 | 337,904 | 340,759 |
| **CPRD** | | | |
|  | **OC within 12 months: Yes** | **OC within 12 months: No** | **Total** |
| **Ovatools ≥3%** | 1,743 (TP) | 10,924 (FP) | 12,667 |
| **Ovatools <3%** | 792 (FN) | 327,300 (TN) | 328,092 |
| **Total** | 2,535 | 338,224 | 340,759 |
| **CPRD + HES APC** | | | |
|  | **OC within 12 months: Yes** | **OC within 12 months: No** | **Total** |
| **Ovatools ≥3%** | 2,230 (TP) | 10,437 (FP) | 12,667 |
| **Ovatools <3%** | 1,061 (FN) | 327,031 (TN) | 328,092 |
| **Total** | 3,291 | 337,468 | 340,759 |
| **CPRD + HES APC + NCRAS** | | | |
|  | **OC within 12 months: Yes** | **OC within 12 months: No** | **Total** |
| **Ovatools ≥3%** | 2,458 (TP) | 10,209 (FP) | 12,667 |
| **Ovatools <3%** | 1,303 (FN) | 326,789 (TN) | 328,092 |
| **Total** | 3,761 | 336,998 | 340,759 |
| **CPRD** - Clinical Practice Research Datalink, **HES APC** - Hospital Episode Statistics**, NCRAS** - National Cancer Registration and Analysis Service, **FN** – False negative, **FP** – False positive, **TN** – True negative, **TP** – True positive | | | |

Table S7: **Distribution of CPRD SNOMED codes among NCRAS confirmed and non-confirmed ovarian cancer cases**

| **SNOMED CT Concept ID** | **Term** | **Confirmed in NCRAS**  **(n)** | **Not confirmed**  **(n)** | **Total**  **(n)** | **Proportion confirmed (%)** |
| --- | --- | --- | --- | --- | --- |
| 128851000 | [m]papillary serous cystadenoma, borderline malignancy | 4 | 0 | 4 | 100 |
| 18854008 | [m]struma ovarii, malignant | 1 | 0 | 1 | 100 |
| 21008007 | [m]cystadenocarcinoma nos | 5 | 0 | 5 | 100 |
| 42194009 | [m]brenner tumour, malignant | 1 | 0 | 1 | 100 |
| 89996007 | [m]brenner tumour, borderline malignancy | 2 | 0 | 2 | 100 |
| 90282004 | [m]papillary serous cystadenocarcinoma | 2 | 0 | 2 | 100 |
| 907041000006102 | [rfc] cancer of the ovary | 3 | 0 | 3 | 100 |
| 90725004 | [m]serous cystadenocarcinoma, nos | 1 | 0 | 1 | 100 |
| 363443007 | malignant neoplasm of ovary | 1447 | 287 | 1734 | 83.4 |
| 128849004 | [m]serous cystadenoma, borderline malignancy | 12 | 3 | 15 | 80 |
| 417629008 | [m]ovarian stromal tumour | 16 | 5 | 21 | 76.2 |
| 115217007 | [M]Ovarian serous tumour | 136 | 51 | 187 | 72.7 |
| 428322007 | malignant neoplasm of ovary and other uterine adnexa | 8 | 3 | 11 | 72.7 |
| 15674004 | [m]serous surface papillary carcinoma | 4 | 2 | 6 | 66.7 |
| 79143006 | [m]mucinous cystadenocarcinoma nos | 4 | 3 | 7 | 57.1 |
| 94974001 | neoplasm of uncertain behaviour of ovary | 8 | 6 | 14 | 57.1 |
| 128852007 | [m]mucinous cystadenoma, borderline malignancy | 2 | 2 | 4 | 50 |
| 67182003 | [m]borderline mucinous cystadenoma of the ovary | 39 | 93 | 132 | 29.5 |
| 363444001 | malignant neoplasm of fallopian tube | 9 | 25 | 34 | 26.5 |
| 30289006 | [m]endometrioid carcinoma | 17 | 123 | 140 | 12.1 |
| 123844007 | [m]endometrioid adenomas and carcinomas | 4 | 35 | 39 | 10.3 |
| 2962009 | [m]cystadenofibroma nos | 4 | 59 | 63 | 6.3 |
| 18105004 | malignant endometrioid adenofibroma | 0 | 1 | 1 | 0 |
| 68880006 | [m]papillary mucinous cystadenocarcinoma | 0 | 1 | 1 | 0 |
| 77029009 | [m]juvenile granulosa cell tumour | 0 | 1 | 1 | 0 |

Table S8: **Distribution of HES APC ICD-10 codes among NCRAS confirmed and non-confirmed ovarian cancer cases**

| **ICD 10** | **Term** | **Confirmed in NCRAS**  **(n)** | **Not confirmed (n)** | **Total**  **(n)** | **Proportion confirmed (%)** |
| --- | --- | --- | --- | --- | --- |
| C56 | Malignant neoplasm of ovary | 1744 | 440 | 2184 | 79.9 |
| C48.2 | Malignant neoplasm: Peritoneum, unspecified | 146 | 43 | 189 | 77.2 |
| C48.1 | Malignant neoplasm: Specified parts of peritoneum | 54 | 23 | 77 | 70.1 |
| D39.1 | BORDER Neoplasm of uncertain or unknown behaviour: Ovary | 162 | 153 | 315 | 51.4 |
| C57.0 | Malignant neoplasm: Fallopian tube | 22 | 61 | 83 | 26.5 |
